# *In Vitro* Uptake of 140 kDa *Bacillus thuringiensis* Nematicidal Crystal Proteins by the Second Stage Juvenile of *Meloidogyne hapla*

**DOI:** 10.1371/journal.pone.0038534

**Published:** 2012-06-21

**Authors:** Fengjuan Zhang, Donghai Peng, Xiaobo Ye, Ziquan Yu, Zhenfei Hu, Lifang Ruan, Ming Sun

**Affiliations:** State Key Laboratory of Agricultural Microbiology, College of Life Science and Technology, Huazhong Agricultural University, Wuhan, China; Universidade de São Paulo, Brazil

## Abstract

Plant-parasitic nematodes (PPNs) are piercing/sucking pests, which cause severe damage to crops worldwide, and are difficult to control. The cyst and root-knot nematodes (RKN) are sedentary endoparasites that develop specialized multinucleate feeding structures from the plant cells called syncytia or giant cells respectively. Within these structures the nematodes produce feeding tubes, which act as molecular sieves with exclusion limits. For example, *Heterodera schachtii* is reportedly unable to ingest proteins larger than 28 kDa. However, it is unknown yet what is the molecular exclusion limit of the *Meloidogyne hapla*. Several types of *Bacillus thuringiensis* crystal proteins showed toxicity to *M. hapla*. To monitor the entry pathway of crystal proteins into *M. hapla*, second-stage juveniles (J2) were treated with NHS-rhodamine labeled nematicidal crystal proteins (Cry55Aa, Cry6Aa, and Cry5Ba). Confocal microscopic observation showed that these crystal proteins were initially detected in the stylet and esophageal lumen, and subsequently in the gut. Western blot analysis revealed that these crystal proteins were modified to different molecular sizes after being ingested. The uptake efficiency of the crystal proteins by the *M. hapla* J2 decreased with increasing of protein molecular mass, based on enzyme-linked immunosorbent assay analysis. Our discovery revealed 140 kDa nematicidal crystal proteins entered *M. hapla* J2 via the stylet, and it has important implications in designing a transgenic resistance approach to control RKN.

## Introduction

Plant-parasitic nematodes (PPNs) are the primary pathogens of potato, sugar beet, soybean, tomato and other crops [1], and cause an estimated annual economic loss of $125 billion worldwide [2]. This damage is mainly caused by cyst nematodes (*Heterodera and Globodera* spp) and root-knot nematodes (*Meloidogyne* spp.). Both *Meloidogyne hapla* and *Meloidogyne incognita* are highly destructive root-knot nematode species, and their genomes have been sequenced [3]. Both these groups of nematodes are sedentary endoparasites and are difficult to control. They live underground and spend most of their lives in the roots, which can offer protection against chemical nematicides [1]. While chemical nematicides remain the most current means of controlling root-knot nematodes [4], their use is declining, because of their toxic effects towards humans and the environment [5].

*Bacillus thuringiensis* is a rod-shaped, Gram-positive, spore-forming bacterium that forms parasporal crystals during the stationary phase of growth [6]. The crystal proteins produced by some of *B. thuringiensis* are pore-forming toxins which are lethal against insects and some nematodes [7], [8]. Nematicidal activity has been found in several families of *B. thuringiensis* crystal proteins, such as Cry5, Cry6, Cry12, Cry13, Cry14, Cry21, and Cry55 [9]. Li et al. reported that Cry6A expressed in transgenic roots significantly impaired the ability of *M. incognita* to reproduce [10]. In addition, a truncated 79 kDa Cry5B expressed in transgenic roots significantly reduced the number of *M. incognita* galls and reduced progeny levels by nearly 3-fold [1]. Until now, our group has isolated several specific *B. thuringiensis* strains which showed high activity against plant-parasitic nematodes [9], [11]. Subsequently, three nematicidal crystal protein encoding genes, *cry6Aa2, cry5Ba2*, and *cry55Aa1*, were isolated from the highly nematicidal *B. thuringiensis* strain YBT-1518 [9] (Table 1). Bioassay results showed that these three crystal proteins were highly toxic to second-stage juveniles (J2) of *M. hapla* [9], and a combination of Cry6Aa and Cry55Aa showed significant synergistic toxicity against *M. incognita* [12].

**Table 1:** The information of Cry55Aa, Cry6Aa, and Cry5Ba used in this study.

Strain	Crystal proteins	Molecular mass	Susceptible host	Source
BMB0250	Cry55Aa	45 kDa	*M. hapla*, *M. incognita*, and *C. elegans*	[9], [12]
BMB0215	Cry6Aa	54 kDa	*M. hapla*, *M. incognita*, and *C. elegans*	[9], [11], [12]
BMB0224	Cry5Ba	140 kDa	*M. hapla*, *M. incognita*, and *C. elegans*	[9], [12]

Plant-parasitic nematodes (PPNs) feed using a specialized stylet. During feeding a tube is produced that acts as a sieve which can only permit the proteins of particular size and dimension to enter the nematode [13]. In beet cyst nematode *Heterodera schachtii* this has been found to be 28 kDa and is referred to as the exclusion limit [14]. However, to date, the exclusion limit of *B. thuringiensis* crystal proteins entering root-knot nematodes have not been reported. Investigating whether or not crystal proteins can enter root-knot nematodes would help to define the molecular exclusion limit and would facilitate the design of a transgenic resistance approach to control root-knot nematodes [14]. In this study, we monitored the pathway of *B. thuringiensis* crystal proteins entering *M. hapla* J2 by confocal laser scanning microscopy (CLSM). Then we detected the changes in the molecular mass of crystal proteins entered *M. hapla* J2 by Western blot. While, the uptake efficiency of the crystal proteins by the *M. hapla* J2 was tested by enzyme-linked immunosorbent assay analysis (ELISA).

## Results

### Use of Resorcinol to Improve *B. thuringiensis* Crystal Protein Efficacy

The previous bioassays used to assess crystal proteins targeting *M. hapla* were conducted with the addition of tomato root exudates (TRE), which potentially increases the frequency of stylet thrusting [15], [16]. In addition, resorcinol stimulates the uptake of double stranded ribonucleic acid (dsRNA) during *in vitro* RNA interference (RNAi) for *M. incognita* J2 [17]. To monitor the role of resorcinol during this bioassay, different concentrations of resorcinol were evaluated to assess its toxicity against *M. hapla* and its effects on stylet thrusting frequency stimulation (Data not shown). The optimum final concentration of resorcinol was determined to be 1 µg/ml.

**Figure 1:**
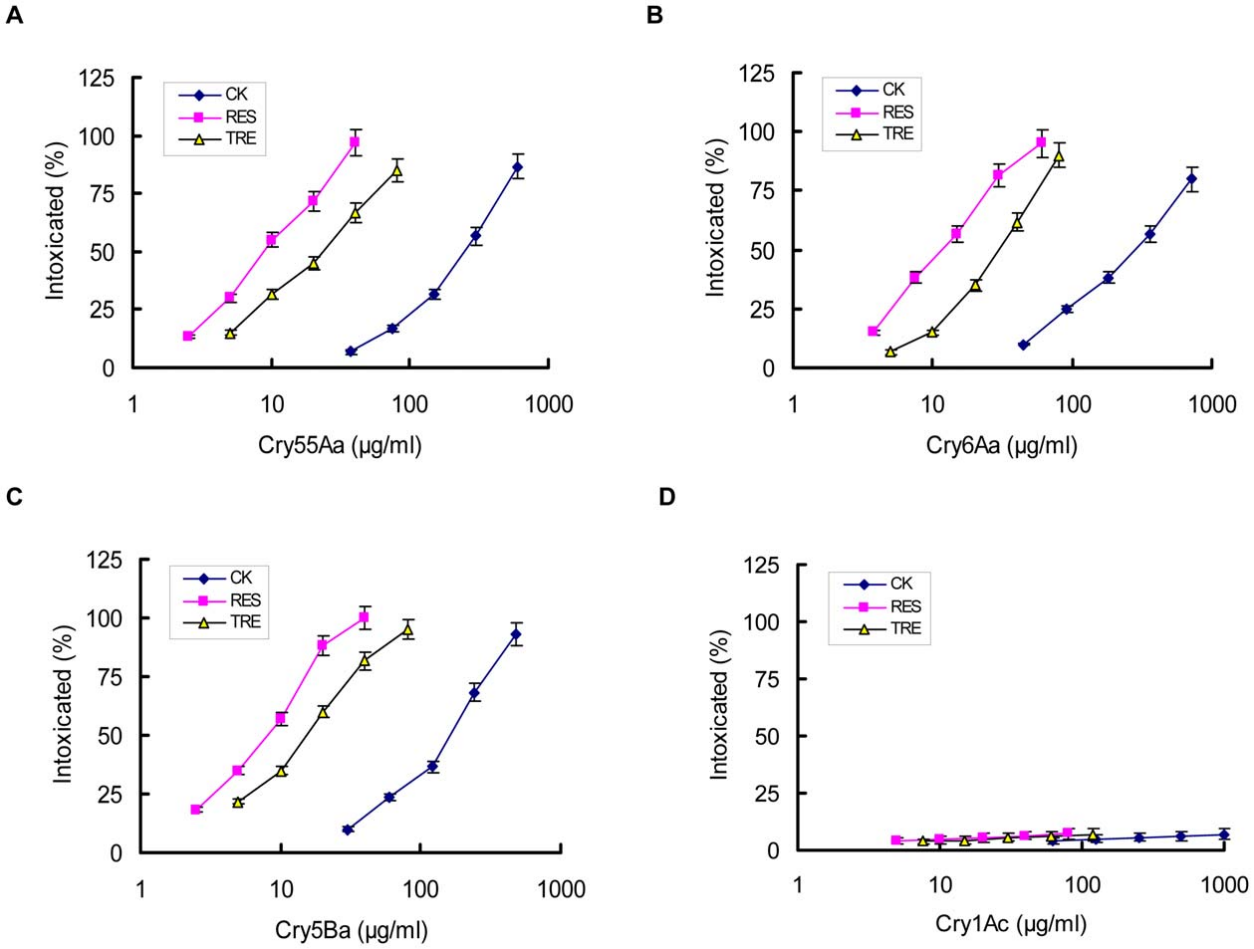
The dose response curves of Cry6Aa, Cry55Aa, and Cry5Ba against *M. hapla* J2 in the presence of resorcinol (RES) or tomato root exudates (TRE). The bioassay of three nematicidal crystal protein Cry6Aa (A), Cry55Aa (B), Cry5Ba (C) against *M. hapla* J2 were conducted in the presence of resorcinol (RES), or tomato root exudates (TRE), or ddH_2_O (CK), respectively. A non-nematicidal crystal protein Cry1Ac (D) was treated as the same and used as control. The *M. hapla* J2 were exposed to five doses of each crystal proteins. Data shown represent the percentage of animals that were intoxicated when fed crystal proteins. Error bars represent the S.D. from the mean of averages over three independent experiments. Each data point represents the average size of 60 animals. The mortality was 3.3% in the absence of any toxins.

For Cry55Aa, the dose at which the intoxicated (%) is reduced to 50% is 10.0 µg/ml in resorcinol, 25.2 µg/ml in TRE, 261.3 µg/ml in ddH_2_O. For Cry6Aa, it is 13.2 µg/ml in resorcinol, 32.6 µg/ml in TRE, 302.1 µg/ml in ddH_2_O. For Cry5Ba, it is 7.6 µg/ml in resorcinol, 16.1 µg/ml in TRE, 156.3 µg/ml in ddH_2_O (Figure 1). These data indicate that, compared with TRE, resorcinol improved the nematicidal activity of crystal proteins in our *M. hapla* bioassay.

### NHS-rhodamine Labeled *B. thuringiensis* Crystal Proteins with Different Molecular Mass (45–140 kDa) can Enter *M. hapla* J2 via the Stylet

**Figure 2:**
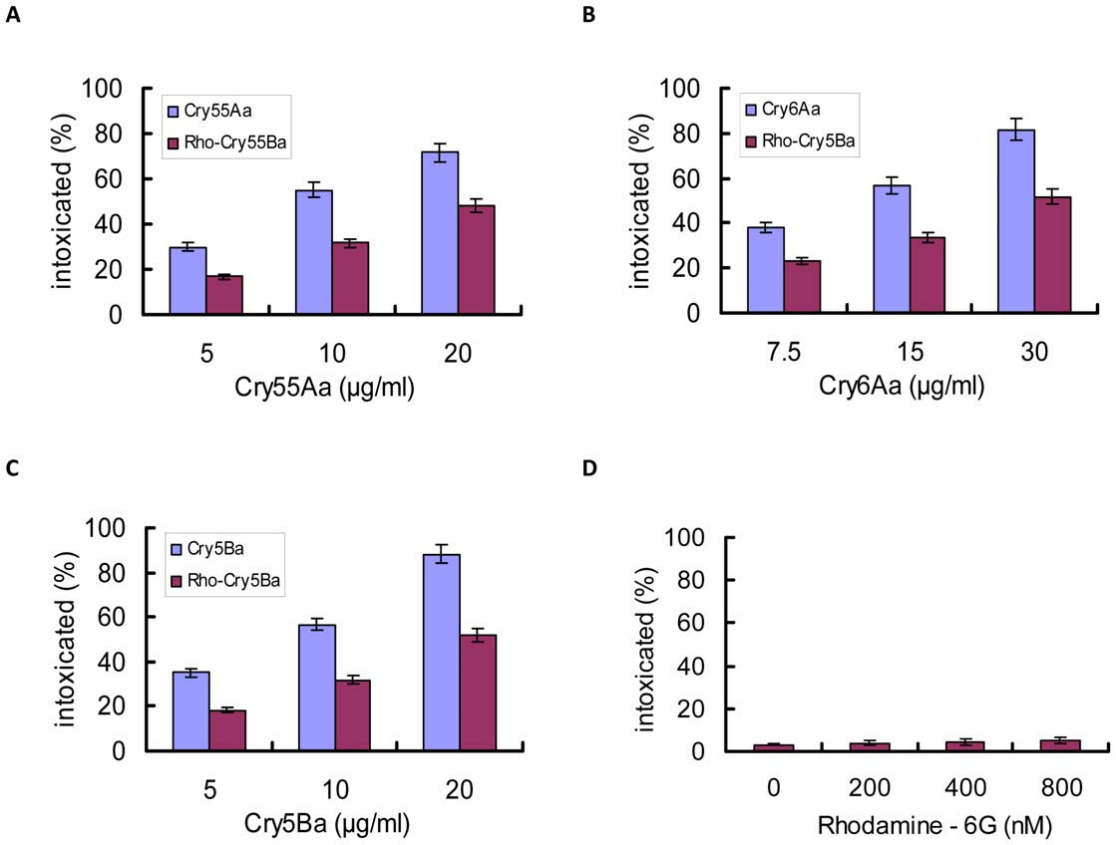
Activities of rhodamine labeled Cry6Aa, Cry55Aa, and Cry5Ba against *M. hapla* J2. *M. hapla* J2 were exposed to three doses of non-labeled crystal protein or rhodamine labeled Cry6Aa (A), Cry55Aa (B), Cry5Ba (C), and rhodamine-6G (D) in the presence of resorcinol. *M. hapla* J2 were exposed to three doses of crystal protein. Data shown represent the percentage of animals that were intoxicated when fed crystal proteins or rhodamine labeled crystal protein. Each data point represents the average size of 60 animals. Error bars represent the S.D. from the mean of averages over three independent experiments.

To confirm the entry pathway of nematicidal crystal proteins, *M. hapla* J2 were incubated in rhodamine-labeled crystal proteins for different periods of time. To confirm whether the rhodamine labeled crystal proteins were active proteins, *M. hapla* J2 were exposed to crystal protein and rhodamine labeled crystal protein respectively in the presence of resorcinol. We found that rhodamine labeled Cry55Aa, Cry6Aa, and Cry5Ba has reduced toxicity to *M. hapla* J2 compared with the non-labeled crystal proteins (Figure 2). The rhodamine 6G was used as a control and it showed no toxicity to *M. hapla* J2 even at the concentration of 800 nM (Figure 2D).

**Figure 3:**
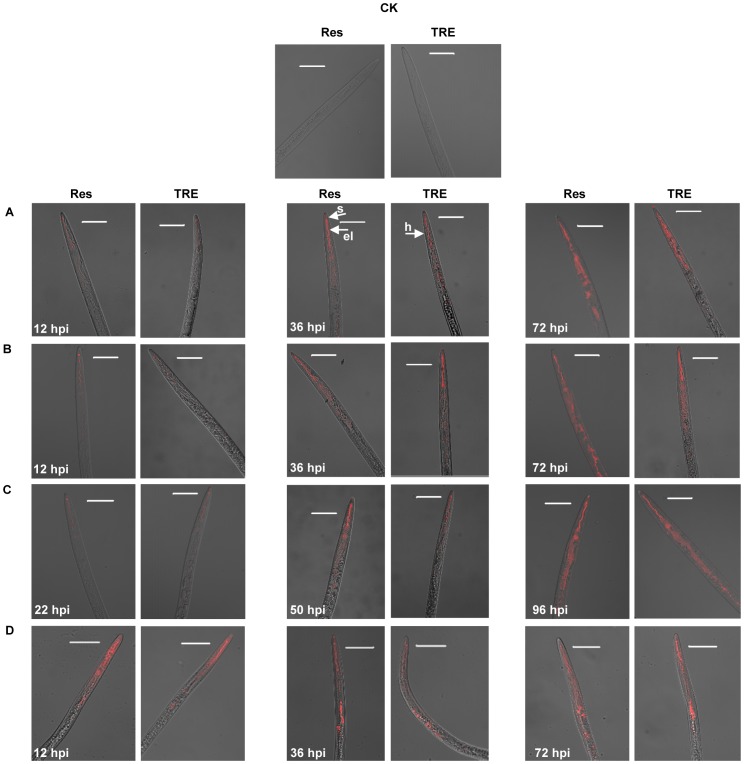
The pathway of nematicidal crystal proteins entering *M. hapla* J2. The confocal laser scanning microscope image showed the ingestion manner and process of Cry55Aa (A), Cry6Aa (B), or Cry5Ba (C) by *M. hapla* J2 in the presence of resorcinol (Res) or tomato root exudates (TRE). *M. hapla* J2 were incubated in rhodamine-labeled crystal toxins for three different times then imaged using a merged image. The rhodamine 6G (D) was treated as the same and used as a control. Toxin was detected inside the treated *M. hapla* J2, but not in the control (CK). The anterior of *M. hapla* is positioned within the upper region. Abbreviation: s = stylet; el = esophageal lumen; h = head of *M. hapla* J2. The scale bar of all the images is 40.43 µm.

The signals from rhodamine-labeled crystal proteins were then monitored by CLSM. *M. hapla* J2 fed with rhodamine 6G (400 nM) alone were used as control. The results are shown in Figure 3, Figure S1 and Figure S2. The photographs were captured under fluorescence illumination (left), bright-field (middle), and merge (right). Due to the molecular exclusion limits of the nematode, two smaller nematicidal crystal proteins Cry55Aa (45 kDa) and Cry6Aa (54 kDa) were initially selected to detect their entry pathway. CLSM showed that Cry55Aa were initially detected in the stylet and esophageal lumen at 12 hours post ingested (hpi), and subsequently in the gut from 36 to 72 hpi in the presence of resorcinol (Figure S1A) or TRE (Figure S2A). The movement of the Cry6Aa toxin through *M. hapla* (Figure S1B and Figure S2B) was identical to that for Cry55Aa. Rhodamine 6G alone was detected in the stylet, esophageal lumen and gut of *M. hapla* J2 at 12 hpi, and the fluorescence was more apparent in gut from 36 to 72 hpi (Figure S1D and Figure S2D). These observations demonstrated that the smaller molecular mass proteins Cry55Aa and Cry6Aa could enter *M. hapla* J2 via the stylet.

To test whether larger molecular mass nematicidal crystal proteins could enter *M. hapla*, similarly experiments were performed by using Cry5Ba (140 kDa). CLSM showed that Cry5Ba were initially detected in the stylet and esophageal lumen at 22 hpi, and subsequently in the gut from 50 to 96 hpi in the presence of resorcinol (Figure S1C) or TRE (Figure S2C). These results demonstrated that the larger molecular mass proteins Cry5Ba could also enter *M. hapla* J2 via the stylet.

### The Molecular Mass of Nematicidal Crystal Proteins become Larger After Ingested by *M. hapla* J2

To monitor the changes in the nematicidal crystal proteins after ingestion, *M. hapla* J2 were fed purified Cry6Aa, Cry55Aa, and Cry5Ba proteins in the presence of resorcinol at different times. Total proteins were then extracted from crystal protein treated nematodes, separated by SDS-PAGE, and subjected to Western blot analysis using an anti-crystal proteins antibody.

**Figure 4:**
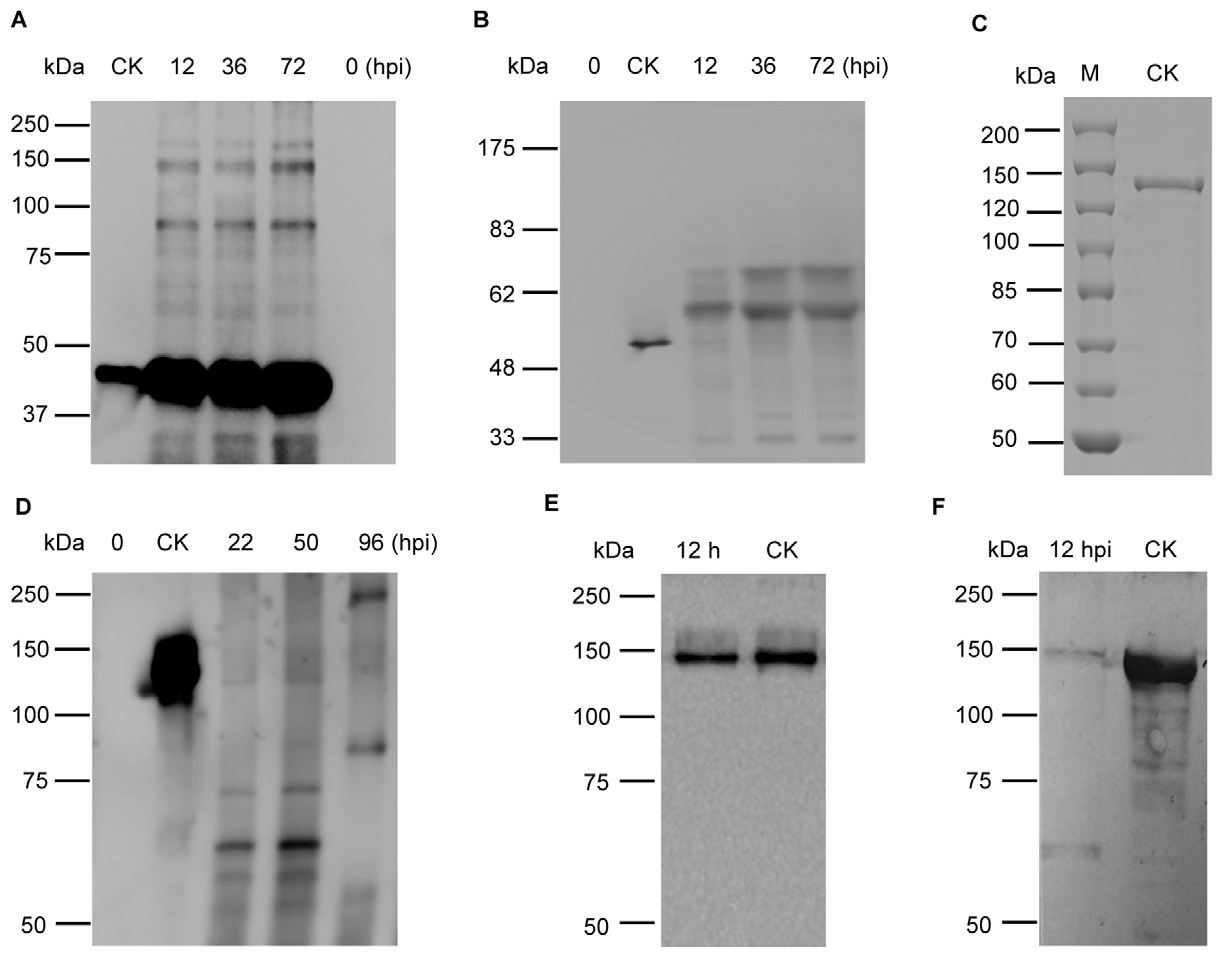
Detection of size changes of Cry55Aa, Cry6Aa, and Cry5Ba in *M. hapla* J2 by Western blot analysis. *M. hapla* J2 were incubated with Cry55Aa protein (Panel A) and Cry6Aa protein (Panel B), and then detected by Western blot at 0, 12, 36, and 72 hpi, using an anti-crystal antibody; *M. hapla* J2 were incubated with Cry5Ba protein (Panel D, F) and then detected by Western blot at 0, 12, 22, 50, and 96 hpi, using Cry5Ba protein antibody. Line CK: Controls of crystal protein without being incubated by *M. hapla* J2. Panel C: SDS-PAGE showed the molecular mass of purified Cry5Ba. Panel E: the Cry5Ba was incubated by *M. hapla* J2 for 12 h, centrifuged at 12000 rpm for 10 min to remove *M. hapla* J2, and then detected by Western blot using Cry5Ba antibody.

Western blot revealed that the molecular mass of Cry6Aa became larger (Figure 4B), approximately 60-kDa at 12 hpi and 70-kDa at 36 hpi. Similarly, the molecular mass of Cry55Aa became larger as well (Figure 4A), in addition to the main 45-kD signal band, signal bands corresponding to approximately 90-kD and 150-kD from 12 hpi till to 72 hpi were observed. The Cry5Ba was also modified after being ingested by *M. hapla* J2. An approximately 60-kDa band was observed between 22 hpi and 50 hpi. The main band subsequently increased to about 90-kDa and 250-kDa at 96 hpi (Figure 4D). To determine whether the 60-kDa Cry5Ba toxin formed before or after ingestion, total proteins were extracted from treated *M. hapla* at 12 hpi, an earlier time than the former 22 hpi. Western blot results indicated a 140 kDa band was present at this time (Figure 4F), suggesting that a 140 kDa form of Cry5Ba entered *M. hapla* J2 directly through the stylet. Based on the above information, we concluded that *M. hapla* J2 can ingest 140 kDa proteins.

### Uptake Efficiency of Crystal Proteins by the *M. hapla* J2 Stylet Decreases with Increasing Protein Molecular Mass

**Figure 5:**
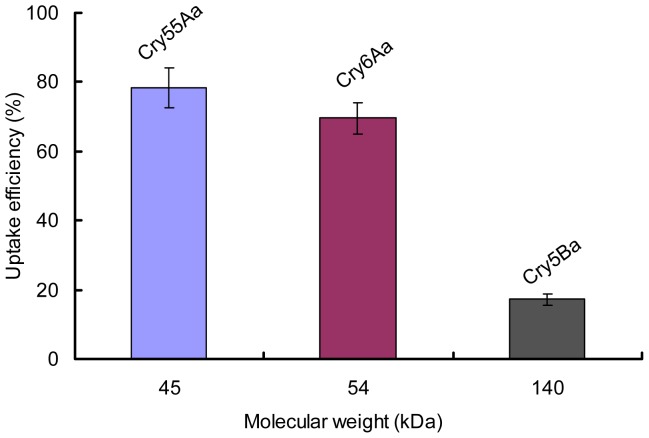
Enzyme-linked immunosorbent assay analysis of the uptake efficiency of nematicidal crystal proteins by *M. hapla* J2. The uptake efficiency was determined by subtracting the percentage crystal proteins uptake in the absence of resorcinol from that in the presence of resorcinol. Each bar value represents the mean SD of triplicate experiments.

The time, taken for *B. thuringiensis* crystal proteins to enter the stylet and esophageal lumen or move into the nematode gut, was different between small and large molecular mass crystal proteins (Figure S1, Figure S2, and Figure 3). We further confirmed the relationship between uptake efficiency and molecular mass of crystal proteins by feeding *M. hapla* J2 with 1500 ng/ml purified Cry55Aa, Cry6Aa, and Cry5Ba for 96 h. The protein concentration after ingestion was then tested by ELISA. The calculated uptake efficiency by *M. hapla* J2 of Cry55Aa (45 kDa), Cry6Aa (54 kDa), and Cry5Ba (140 kDa) proteins were 78.3%, 69.5%, and 17.2%, respectively (Figure 5). These data showed the uptake efficiency of crystal proteins by *M. hapla* J2 stylet decreased with increasing protein molecular mass.

## Discussion

The original bioassay for the detection of crystal proteins targeting *M. hapla* was conducted with the addition of TRE [9]. TRE can attract nematodes to plant roots, induce stylet thrusting, release of secretions and increase in nematode mobility [16]. In this study, we improved the *B. thuringiensis* crystal proteins bioassay protocol for *M. hapla* J2 by using resorcinol instead of TRE. Resorcinol was previously used to stimulate the uptake of dsRNA during *in vitro* RNAi of *M. incognita* J2 [17]. Compared with TRE, resorcinol is simple and more stable, and may improve the crystal nematicidal activity during the *M. hapla* bioassay (Figure 1).

The pathway of *B. thuringiensis* crystal proteins entering *M. hapla* is still not clear. Cuticle penetration is predominately believed to be the primary action mode of the extracellular proteases during nematode infection [18]. For example, *Brevibacillus laterosporus* secretes extracellular proteases that damage the nematode cuticle [18], [19]. Initially we conjectured that crystal proteins were able to enter *M. hapla* J2 through nematode cuticle. However, in this *in vitro* study, our results confirmed that *M. hapla* J2 could ingest a range of proteins sizes from 45 kDa to 140 kDa directly through the stylet (Figure 3 and Figure 4). When the head of *M. hapla* J2 was magnified, the signal of Cry5Ba around stylet and esophageal was also detected, but it was weaker in comparison with that in stylet and esophageal lumen (Figure S1C1 and Figure S1C2). So Cry protein could enter mainly through the stylet, it can also enter through the mouth and flowing around the stylet but still entering. Also, we found the uptake efficiency of crystal proteins by *M. hapla* J2 decreased with increasing protein molecular mass. This situation was similar to the previous reports that fluorescent molecule diffusion speed in the syncytium was dependent upon its size [13]. About the uptake size of *M. hapla* J2, We found 140 kDa Cry5B can enter *M. hapla* J2 and the uptake efficiency was very low (17.2%). However, we did not assess proteins larger than 140 kDa, maybe larger proteins could also enter *M. hapla* J2 stylet.

It’s known that the secretions of cyst, root-knot and a few other sedentary endoparasitic nematodes produced a feeding tube at the interface between the syncytial cytoplasm and the nematode’s stylet [20], [21]. Differences in molecular exclusion limits of the cyst nematode *H. schachtii* and the root-knot nematodes may be due to the variation in the ultra-structure and size of their feeding tubes [4]. It is reported that the beet cyst nematode *H. schachtii* was unable to ingest proteins larger than 28 kDa [14]. Goverse *et* *al.* reported that *Globodera rostochiensis* juveniles could ingest 32 kDa proteins [22]. While, Cry6A and a truncated 79 kDa Cry5B expressed in transgenic roots significantly impaired the ability of *M. incognita* to reproduce [1], [10], indicating the feeding tube of *M. incognita* can uptake a protein of 79 kDa *in vivo*.

In the experimental system described here, we demonstrated that different sized *B. thuringiensis* crystal proteins can enter *M. hapla* J2 through the stylet. Although this *in vitro* experiment may not be applicable to feeding tubes produced *in vivo*, it would suggest that 140 kDa cry proteins if produced as extracellular secreted protein by a transgenic plant could be taken up by the nematode. Stylet thrusting is a natural phennomenon induced by TRE. One could imagine that during the migrating phase of the J2 within the root system it is exposed to extracellular root secretions most if not all of which are also present in TRE. Therefore, it can be envisaged that cry proteins can be expressed as extracellular plant secretions. Our discovery has important implications in controlling *M. hapla* J2 during the migratory phase of the second stage juvenile before the J2 becomes sedentary and sets up its feed site and the subsequent formation of its feeding tube.

In summary, we demonstrated in an *in vitro* system that 140 kDa *B. thuringiensis* crystal proteins can enter *M. hapla* J2 through the stylet. It has important implications for the design of any transgenic resistance approach against *M. hapla*.

## Materials and Methods

### Ethics Statement

All the procedures related to animal housing, handling, care and treatment in this study were approved by the Laboratory Animal Monitoring Committee of Hubei province of China and performed accordingly, the approval ID: SYXK 2005–0029.

### Bacterial Strains and Media

*B. thuringiensis* strains BMB0250, BMB0224, and BMB0215 [9] were used for the preparation of nematicidal crystal proteins Cry55Aa, Cry5Ba, and Cry6Aa, respectively. All *B. thuringiensis* strains were maintained on Luria-Bertani (LB) agar plates and supplemented with appropriate antibiotics at 28°C [23].

### *M. hapla* Rearing and Bioassay

The *M. hapla* bioassay procedure was undertaken according to the method described by Bischof et al [24]. The toxicity of crystal proteins against *M. hapla* J2 was tested by touching the worms directly, typically 3 times or so, and then looking for motility. A visibly moving nematode was marked as alive. Nematodes that were not moving were gently touched with a platinum pick and watched for movement. Nematodes that failed to respond after several touches were marked as dead or intoxicated [24].

Sixty *M. hapla* J2 larvae were individually placed into each well of 96-well microtiter plates (Corning, 3513). Resorcinol (1 µg/ml) [17] or TRE [15], [16] was used to induce an increase in stylet pulsing frequency. Cry1Ac was used as the control. The dose at which the intoxicated (%) is reduced to 50% of the crystal toxin against *M. hapla* J2 was evaluated using SAS 8.0 software.

### Crystal Protein Purification and Labeling

Cry55Aa, Cry5Ba, and Cry6Aa proteins were purified according to the method described by Guo et al. [9]. All purified protein samples were then solubilized in 20 mM HEPES (Calbiochem BB0364) (pH 8.0), quantified [25], and stored at −80°C. Purified proteins were labeled with N-hydroxysuccinimide–rhodamine (Pierce 46102) according to the method described by Griffitts et al. [26].

### Confocal Laser Scanning Microscope (CLSM) Detection

*M. hapla* were incubated in M9 medium [27] containing 2 µg/ml proteins for each time period and washed five times in M9 medium before confocal laser scanning microscopy (CLSM; Zeiss LSM 510) imaging [28]. Resorcinol (1 µg/ml) or TRE was added to induce stylet pulsing frequency. Images were captured using a 40×objective. Fluorescence was monitored at an excitation wave-length of 543 nm and a high pass filter (LP 560). Images were merged and data were stored.

### Western Blot Analysis

*M. hapla* treated with purified crystal proteins for different times were harvested and subsequently washed five times in M9 medium [28]. Treated *M. hapla* samples were grinded with liquid nitrogen, and then subjected to sodium dodecyl sulfate polyacrylamide gel electrophoresis (SDS-PAGE) and Western blot analysis [10]. Antisera of Cry55Aa, Cry5Ba, and Cry6Aa were prepared according to the following procedure: purified crystal proteins were separated by SDS-PAGE and the gels were stained and destained. Purified crystal proteins bands were then excised from the gel, washed three times with water for 5 min each time, and used to immunize rabbits for antibody development according to standard protocols [29]. The protocol used to immunize the rabbits was described in the supporting method (see Protocol S1).

### ELISA Analysis

Enzyme-linked immunosorbent assay (ELISA) was conducted according to the protocol described by Huang et al. [17] with some modifications. ELISA plates were incubated at 4°C for 12 h with different concentrations of crystal proteins in 20 mM HEPES (pH 8.0) and washed five times with PBST (135 mM NaCl, 2 mM KCl, 10 mM Na_2_HPO_4_, 1.7 mM KH_2_PO_4_, pH 7.5, 0.1% Tween-20). Plates were then blocked in 200 µl PBST plus 2% BSA for 2 h at room temperature (RT), and washed five times with PBST. ELISA plates were incubated with anti-crystal protein antibody (1∶1000) for 2 h at RT, followed by a secondary goat-anti-rabbit-horseradish peroxidase (HRP) antibody for 2 h at RT. The HRP enzymatic activity was determined using a freshly prepared substrate (100 µl TMB) at RT for 40 min. Then the enzymatic reaction was stopped with 100 µl 2 M H_2_SO_4_, and the absorbance was read at 450 nm.

### Detection of Crystal Proteins Uptake Efficiency

*M. hapla* J2 were fed on 1500 ng/ml purified crystal proteins in the presence or absence of resorcinol for 96 h. Crystal protein concentration after ingestion was determined from standard curves by ELISA. Uptake efficiency was determined by subtracting crystal proteins uptake percentage in the absence of resorcinol from that in the presence of resorcinol.

## Supporting Information

Figure S1 **The pathway of nematicidal crystal proteins entering** ***M. hapla*** **J2 in the presence of resorcinol.** Confocal laser scanning microscope image showing ingestion of Cry55Aa (A), Cry6Aa (B), Cry5Ba (C), or rhodamine 6G (D) in treated *M. hapla* J2 in the presence of resorcinol. *M. hapla* J2 were incubated in rhodamine-labeled crystal toxins for three different times, then imaged using the bright-field to visualize the *M. hapla* (Middle), the rhodamine channel to visualize toxin (Left) and merged image (Right). Toxin was detected inside the treated *M. hapla*, but not in the control (CK). The anterior of *M. hapla* is positioned within the upper region. s = stylet; el = esophageal lumen; h = head of *M. hapla* J2; C1 and C2: the magnification of head of *M. hapla* J2. The scale bar of C1 and C2 is 5.93 µm. The scale bar of other images is 40.43 µm.
(TIF)

Figure S2 **The pathway of nematicidal crystal proteins entering** ***M. hapla*** **J2 in the presence of tomato root exudates.** Confocal laser scanning microscope image showing ingestion of Cry55Aa (A), Cry6Aa (B), Cry5Ba (C), or rhodamine 6G (D) in treated *M. hapla* J2 in the presence of tomato root exudates. *M. hapla* J2 were incubated in rhodamine-labeled crystal toxins for different times, then imaged using the bright-field to visualize the *M. hapla* (Middle), the rhodamine channel to visualize toxin (Left), and merged image (Right). Toxin was detected inside the treated *M. hapla*, but not in the control (CK). The anterior of *M. hapla* was positioned within the upper region. The scale bar of all the images is 40.43 µm.
(TIF)

Protocol S1 **Supporting Method.** Preparation of antiserum.
(DOC)
