# Supplementary figures and images for: In Vitro Uptake of 140 kDa Bacillus thuringiensis Nematicidal Crystal Proteins by the Second Stage Juvenile of Meloidogyne hapla
Source: PLoS One. 2012 Jun 21;7(6):e38534. doi: 10.1371/journal.pone.0038534 (PMC3380895; doi:10.1371/journal.pone.0038534)

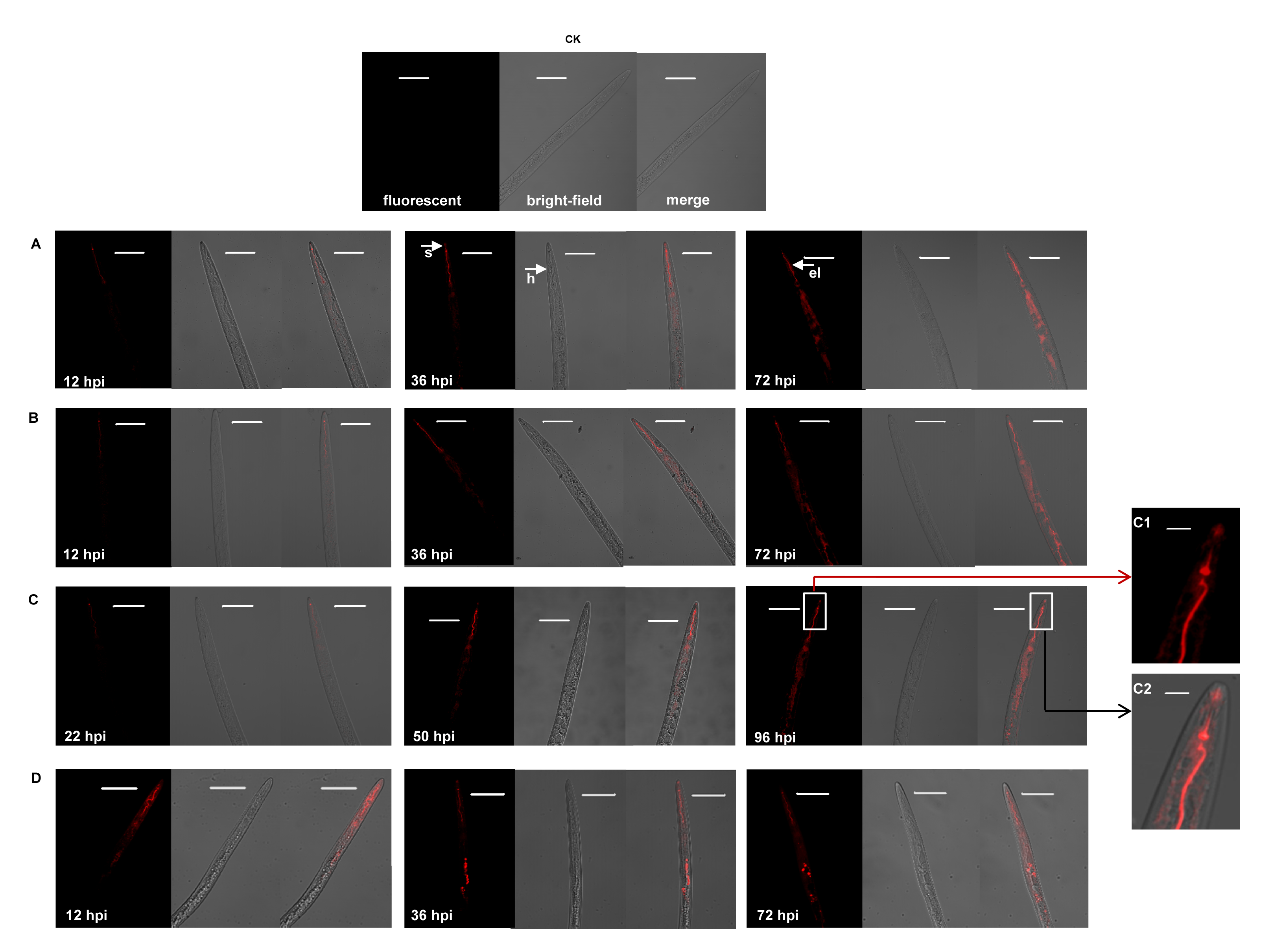

Supplement: Figure S1 — The pathway of nematicidal crystal proteins entering M. hapla J2 in the presence of resorcinol. Confocal laser scanning microscope image showing ingestion of Cry55Aa (A), Cry6Aa (B), Cry5Ba (C), or rhodamine 6G (D) in treated M. hapla J2 in the presence of resorcinol. M. hapla J2 were incubated in rhodamine-labeled crystal toxins for three different times, then imaged using the bright-field to visualize the M. hapla (Middle), the rhodamine channel to visualize toxin (Left) and merged image (Right). Toxin was detected inside the treated M. hapla, but not in the control (CK). The anterior of M. hapla is positioned within the upper region. s = stylet; el = esophageal lumen; h = head of M. hapla J2; C1 and C2: the magnification of head of M. hapla J2. The scale bar of C1 and C2 is 5.93 µm. The scale bar of other images is 40.43 µm. (TIF) [file pone.0038534.s001.tif]

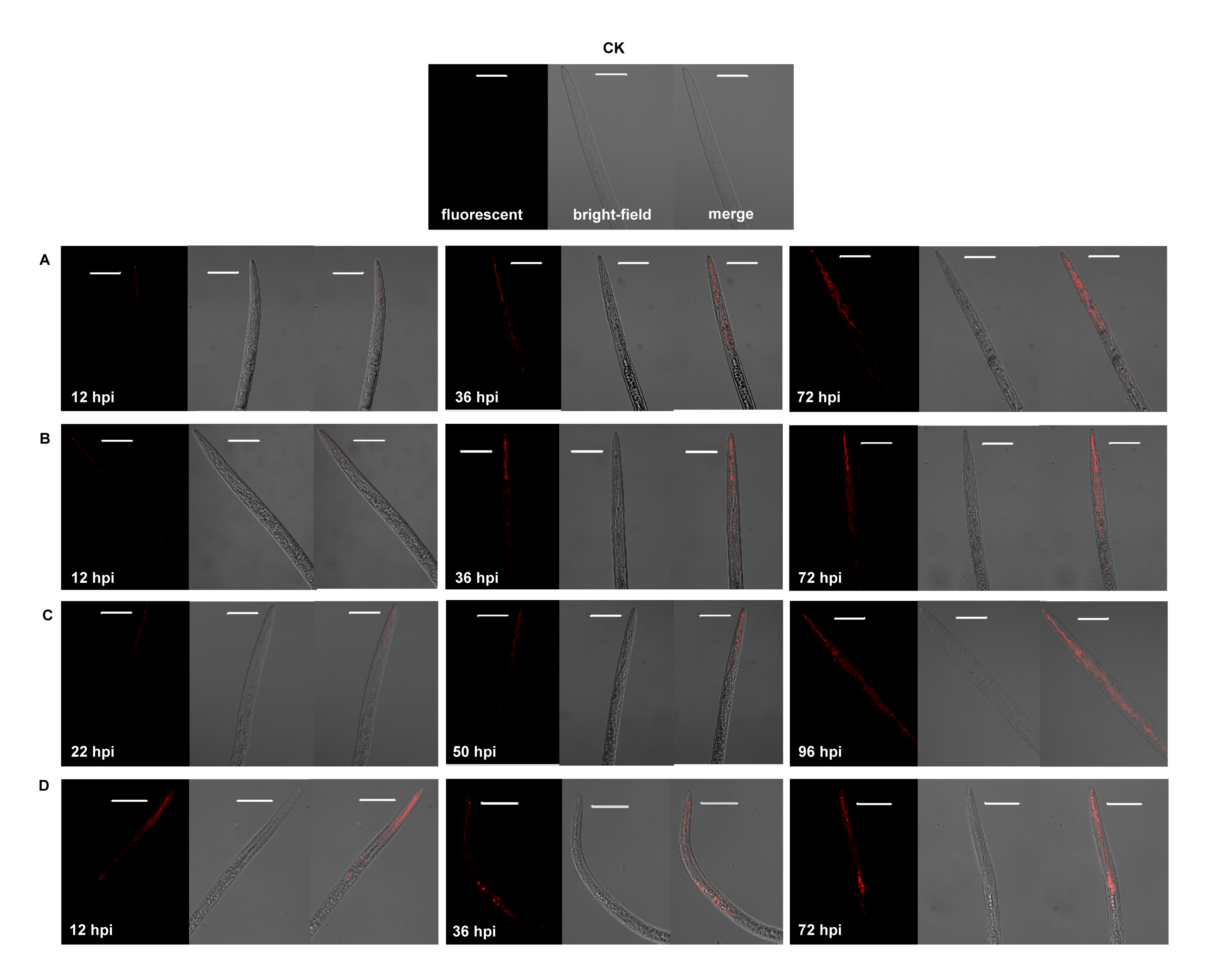

Supplement: Figure S2 — The pathway of nematicidal crystal proteins entering M. hapla J2 in the presence of tomato root exudates. Confocal laser scanning microscope image showing ingestion of Cry55Aa (A), Cry6Aa (B), Cry5Ba (C), or rhodamine 6G (D) in treated M. hapla J2 in the presence of tomato root exudates. M. hapla J2 were incubated in rhodamine-labeled crystal toxins for different times, then imaged using the bright-field to visualize the M. hapla (Middle), the rhodamine channel to visualize toxin (Left), and merged image (Right). Toxin was detected inside the treated M. hapla, but not in the control (CK). The anterior of M. hapla was positioned within the upper region. The scale bar of all the images is 40.43 µm. (TIF) [file pone.0038534.s002.tif]
