# Supplementary material for: In Vitro Uptake of 140 kDa Bacillus thuringiensis Nematicidal Crystal Proteins by the Second Stage Juvenile of Meloidogyne hapla
Source: PLoS One. 2012 Jun 21;7(6):e38534. doi: 10.1371/journal.pone.0038534 (PMC3380895; doi:10.1371/journal.pone.0038534)
Supplement: Protocol S1 — Supporting Method. Preparation of antiserum. (DOC) [file pone.0038534.s003.doc]

***In vitro* uptake of 140 kDa *Bacillus thuringiensis* nematicidal crystal proteins by the second stage juvenile of *Meloidogyne hapla***

Fengjuan Zhang^†^, Donghai Peng^†^, Xiaobo Ye, Ziquan Yu, Zhenfei Hu, Lifang Ruan, and Ming Sun*

**Protocol S1. Supporting Method**

**Preparation of antiserum.** The excised bands were washed three times with water for 5min each time, and then the excised bands were triturated. About 1 ml triturated sample in physiological saline containing 1 mg antigen was mixed with 1 ml Freund’s complete adjuvant, and injected subcutaneously two adult white rabbits. After the initial injection, the rabbits were injected with similar amounts of antigen and adjuvant by the same manner 7 and 14 days later. Finally, a booster intravenous injection was administered 14 days later. Beginning 7 days after the booster injection, 2 ml blood was collected from the ear vein of the immunized rabbit to detect the titer of antibody by ELISA. When the titer of antibody was greater than 1:10000, the blood were collected from the carotid artery of the immunized rabbit.
